# In Vitro Intracellular Hyperthermia of Iron Oxide Magnetic Nanoparticles, Synthesized at High Temperature by a Polyol Process

**DOI:** 10.3390/pharmaceutics12050424

**Published:** 2020-05-06

**Authors:** Cristian Iacovita, Ionel Fizeșan, Anca Pop, Lavinia Scorus, Roxana Dudric, Gabriela Stiufiuc, Nicoleta Vedeanu, Romulus Tetean, Felicia Loghin, Rares Stiufiuc, Constantin Mihai Lucaciu

**Affiliations:** 1 Department of Pharmaceutical Physics-Biophysics, Faculty of Pharmacy, “Iuliu Hatieganu” University of Medicine and Pharmacy, Pasteur 6, 400349 Cluj-Napoca, Romania; cristian.iacovita@umfcluj.ro (C.I.); scorus.lavinia@gmail.com (L.S.); simona.vedeanu@umfcluj.ro (N.V.); 2 Department of Toxicology, Faculty of Pharmacy, “Iuliu Hațieganu” University of Medicine and Pharmacy, Pasteur, 6A, 400349 Cluj-Napoca, Romania; ionel.fizesan@umfcluj.ro (I.F.); ancapp@gmail.com (A.P.); floghin@umfcluj.ro (F.L.); 3 Faculty of Physics, “Babes Bolyai” University, Kogalniceanu 1, 400084 Cluj-Napoca, Romania; roxana.pacurariu@phys.ubbcluj.ro (R.D.); gabi.stiufiuc@phys.ubbcluj.ro (G.S.); romulus.tetean@phys.ubbcluj.ro (R.T.); 4 Department of Bionanoscopy, MedFuture Research Center for Advanced Medicine, “Iuliu Hatieganu” University of Medicine and Pharmacy, Pasteur 4-6, 400337 Cluj-Napoca, Romania

**Keywords:** iron oxide magnetic nanoparticles, polyol synthesis method, magnetic hyperthermia, Alamar Blue assay, Neutral Red assay, A549 cancer cells, HGF normal cells, intracellular hyperthermia, nanoscale heating, lysosomes

## Abstract

We report the synthesis of magnetite nanoparticles (IOMNPs) using the polyol method performed at elevated temperature (300 °C) and high pressure. The ferromagnetic polyhedral IOMNPs exhibited high saturation magnetizations at room temperature (83 emu/g) and a maximum specific absorption rate (SAR) of 2400 W/g_Fe_ in water. The uniform dispersion of IOMNPs in solid matrix led to a monotonous increase of SAR maximum (3600 W/g_Fe_) as the concentration decreased. Cytotoxicity studies on two cell lines (cancer and normal) using Alamar Blues and Neutral Red assays revealed insignificant toxicity of the IOMNPs on the cells up to a concentration of 1000 μg/mL. The cells internalized the IOMNPs inside lysosomes in a dose-dependent manner, with higher amounts of IOMNPs in cancer cells. Intracellular hyperthermia experiments revealed a significant increase in the macroscopic temperatures of the IOMNPs loaded cell suspensions, which depend on the amount of internalized IOMNPs and the alternating magnetic field amplitude. The cancer cells were found to be more sensitive to the intracellular hyperthermia compared to the normal ones. For both cell lines, cells heated at the same macroscopic temperature presented lower viability at higher amplitudes of the alternating magnetic field, indicating the occurrence of mechanical or nanoscale heating effects.

## 1. Introduction

Since the validation of ferrimagnetic iron oxide magnetic nanoparticles, IOMNPs (magnetite—Fe_3_O_4_ or maghemite—Fe_2_O_3_), by the U.S. Food and Drug Administration [1], this class of magnetic nanoparticles has been the subject of intense research for their potentiality in a widespread number of biomedical and pharmaceutical applications [2,3,4,5,6]. To be efficient as vectors for targeted drug delivery, contrast agents in magnetic resonance imaging (MRI) and heating mediators in magnetic hyperthermia (MH), the IOMNPs should possess a maximum value of saturation magnetization (*M*_s_) and minimum value of coercive field (*H*_c_) and remnant magnetization (*M*_r_) [7,8,9]. However, IOMNPs have shown limited values of their magnetic properties, and, as a consequence, they have demonstrated limited heating capabilities [2]. Therefore, the clinical applications of MH based tumor treatments for glioblastoma and prostate cancer were used in conjunction with chemotherapy and radiotherapy [10]. To overcome these limitations, intense research efforts have been devoted to the fabrication of IOMNPs with significantly enhanced heating capabilities by controlling their composition, size, size distribution, shape, and surface coatings [11]. Notwithstanding these efforts, the maximum heating ability of IOMNPs in an alternating magnetic field (AMF) is limited both by their intrinsic physical properties and by the safety limits imposed for the magnetic field parameters (H amplitude of the AMF and f frequency) such as H × f < 5 × 10^9^ Am^−1^s^−1^ [12]. New momentum in IOMNPs-MH research was produced by recent studies suggesting that IOMNPs can significantly raise, at the nanoscale range, the temperature of molecules bound to their surfaces or situated in their proximity, thus leading to changes in their physiological and biochemical properties, which turn into functional changes in the organisms. Among other mechanisms that may explain the biological effects of the nanoscale heating, it was suggested that the accumulation of MNPs in the lysosomes and their subsequent heating under an AMF would also lead to an increase in the generation of reactive oxygen species (ROS) and finally, to cellular death by a Caspase-1 dependent mechanism [13,14]. These effects leading to cellular death need further, more detailed investigations aiming at explaining the actual mechanisms relating the IOMNPs-MH characteristics to the biological effects.

Various synthesis routes have been explored for the production of IOMNPs with enhanced magnetic properties [15,16]. In particular, the non-hydrolytic thermal decomposition of organometallic magnetic precursors in organic solvents with high boiling temperatures has to be mentioned [17] as well as the hydrolytic synthesis methods, namely, co-precipitation [18], hydrothermal treatment [19], and polyol process [20,21]. The latter synthesis technique has been widely used in the last decades, mostly owing to the outstanding inherent properties of polyols employed as the solvent. The high dielectric constants of polyols allow the dissolution of iron(II) or iron(III) inorganic salts (as nitrates, sulfates or chlorides) or acetylacetonates, precipitator compounds (as sodium hydroxide, sodium acetate) as well as different extra stabilizer agents (as polyvinylpyrrolidone, PVP) facilitating the realization of a great variety of reaction mixtures, thus enabling the preparation of a wide range of IOMNPs [22]. Additionally, as proven earlier on the synthesis of both silver and gold nanoparticles [23,24], the polyols might serve as reducing agents as well as stabilizers, controlling the growth of the IOMNPs and assuring their colloidal stability [25]. Consequently, the resulted IOMNPs were coated with hydrophilic and biocompatible polyol ligands and exhibited valuable magnetic properties, thus holding great potential for biomedical applications. Depending on the nature of the polyol solvent, the amount of precipitator, the presence of an extra stabilizer, and the reaction time, the polyol method yielded IOMNPs with well-defined shapes such as spheres, cubes and polyhedrals [25,26,27,28,29,30,31,32,33] with sizes of several nanometers to a few hundreds of nanometers [25,26,27,28,29,30,31,32,33] and controlled morphologies (e.g., flower-like, hollow spheres and nanoclusters [34,35,36,37,38,39]). The later classes of IOMNPs, due to the exchange coupling between the constituents, have shown enhanced relativities and heating efficiency compared to single-core IOMNPs [34,35,36,37,38,39]. Alternatively, spherical single-core IOMNPs displaying enhanced magnetic properties and resulting in improved relaxivity values have been elaborated in an autoclave at high pressure and high-temperature conditions by increasing the synthesis temperature above the boiling point of the polyols employed [40].

In this paper, we have made use of the conventional polyol mediate route, with iron(III) chloride dissolved in PEG200, followed by a solvothermal reaction taking place at 300 °C in the presence of a variable amount of sodium acetate (NaAc), aiming at synthesizing IOMNPs with high heating capabilities. The main goals were to find the optimal preparation of IOMNPs and to reach an intracellular concentration high enough to induce an effective MH heating, but below the concentration values affecting the viability of the cells, in zero field conditions. We analyzed the role of the amount of NaAc in the size and morphology of IOMNPs, the reaction time being reduced to only 1 h. The IOMNPs were coated with citric acid [38], followed by a systematic study of their structural, magnetic, and hyperthermia properties. The IOMNPs providing the best heating performances were further selected and their in vitro hyperthermia performance and their toxicity evaluated. In preliminary studies, we assessed the citric acid-coated IOMNP uptake by two types of cells: a human pulmonary cancer cell line (A549) and a human gingival fibroblasts (HGF) normal cell line. The intracellular hyperthermia effects on the two cell lines were assessed by using two complementary assays: the neutral red (NR)-uptake assay, which is related mainly to lysosomal activity of the cells and the Alamar Blue (AB) assay, which gives more general information related to the whole-cell metabolism. In this way, by comparing the results obtained by these two methods, we tried to bring more insights into the mechanisms of cell death induced by MH as well as through the lysosomal cytotoxicity pathway.

## 2. Materials and Methods

### 2.1. Synthesis

All reagents employed in this study were of analytical grade and were used without any further purification. The iron oxide magnetic nanoparticles (IOMNPs) were synthesized by using the following products: iron(III) chloride hexahydrate (FeCl_3_ 6H_2_O) (Roth, ≥98%), polyethylene glycol 200 (PEG200) (Roth, ≥99%), and sodium acetate trihydrate (NaAc) (Roth, ≥99.5%).

The synthesis of IOMNPs was performed using a polyol mediated synthetic route, as follows: 0.27 g of FeCl_3_ 6H_2_O and a variable amount of NaAc (0–4.8 g) were mixed and dissolved in 40 mL PEG200. The solutions were stirred thoroughly via a magnetic stirrer at 50 °C, 500 rot/min for 30 min, and transferred in a 60 mL round-bottom flask, being part of a home-made stainless steel reaction vessel. Before sealing the reaction vessel using a Teflon gasket and five screws, the solutions were degassed by exposure to a flux of gaseous nitrogen for 5 min. The reaction vessel was introduced into an oven (Nabertherm GmbH, Lilienthal, Germany) equipped with a temperature controller (JUMO dTron 316) that allowed for programming the heating. The solutions were heated from room temperature to 300 °C with a constant heating rate of 3 °C/min and kept at this temperature for 1 h. The vessel was left to cool at room temperature, the excess liquid was discharged, and the obtained black precipitates were washed with ethanol/double distilled water, employing five ultrasonication/magnetic separation cycles to remove the excess ligands and unreacted precursors. The washing cycle consisted of ultrasonication of the black precipitates in 30 mL of ethanol/double distilled water (*v:v* = 1:2) for 15 min followed by magnetic separation using a neodymium magnet. Furthermore, the IOMNPs were coated with citric acid according to a procedure adapted from [38]. Briefly, 20 mg of IOMNPs powder, obtained by drying the black precipitate in a rota-evaporator, was dispersed in a 40 mL aqueous solution of citric acid (c = 0.1 M). Afterward, the solutions were heated at a temperature of 80 °C for 30 min to ensure the grafting of citric acid molecules on the surface of the IOMNPs. Subsequently, the IOMNPs were magnetically separated and washed with double distilled water and re-dispersed in 5 mL of double-distilled water. Finally, the pH of the IOMNPs solutions (c = 4 mg_IOMNPs_/mL) was adjusted to 7 with an aqueous solution of HNO_3_ (c = 0.01 M) for further analysis.

### 2.2. Characterization

Transmission electron microscopy (TEM) analysis was performed on a Hitachi HT7700 (Hitachi Ltd., Tokyo, Japan) setup operating at 100 kV in high contrast mode. An 8-megapixel Charge-Coupled Device (CCD) camera was employed to capture the images of IOMNPs. The TEM samples were prepared by placing a 5 μL drop of the aqueous solution of IOMNPs on carbon-coated copper grids, removing the excess water by filter paper after 2 min and drying them under ambient air.

X-ray diffraction (XRD) measurements were carried out on a Bruker D8 Advance diffractometer using Cu Kα radiation (Bruker AXS GmbH, Karlsruhe, Germany). The diffractograms were recorded at room temperature on a powder of IOMNPs obtained by drying them in a rota-evaporator. The FullProf software (FullProf.2k (Version 7.00-May2019-ILL JRC https://www.ill.eu/sites/fullprof/) was employed to detect the crystalline phases and to calculate the lattice parameters.

Magnetic measurements were performed on powder samples at 300 K in magnetic fields up to 2 T using a vibrating sample magnetometer (VSM) produced by Cryogenic Limited (London, UK).

Magnetic hyperthermia measurements were carried out using the magnetic heating system Easy Heat 0224 provided by Ambrell (Scottsville, NY, USA). According to the calibration presented in [32], the setup was capable of generating alternating magnetic fields (AMF) with strengths between 5 kA/m and 65 kA/m at a maximum frequency of 355 kHz in the center of an 8-turn coil. A fiber-optic probe, placed in the middle of the sample volume, was employed to measure the temperature value each second. The environment in close vicinity of samples was held at a physiological temperature around 37 °C. The samples usually consisted of 0.5 mL of IOMNP suspensions in water and solid PEG8000 at different concentrations. The specific absorption rate (SAR), defined as the heat released from a suspension of MNPs in unit time reported to the mass of iron content, was used to quantify the heat performance of IOMNPs. For reliable determination of SAR, the heating curves were fitted with the Box–Lucas Equation [41]:

(1) $$\Delta T=\frac{S_{m}}{k}\;\left(1-e^{-k\left(t-t_{0}\right)}\right)$$

where the fitting parameters $S_{m}$ and k are the initial slope of the heating curve and the constant describing the cooling rate, respectively. Thus, SAR can be calculated as:

(2) $$SAR=\frac{c\;m\;S_{m}}{m_{Fe}}$$

where c is the specific heat of the colloid (in our case was approximated with the specific heat either of water: $c=4186.8\frac{J}{\mathrm{kg}\;K}$ or of PEG8k: $c=2135.27\;\frac{J}{\mathrm{kg}\;K}$, the IOMNPs contribution to the specific heat being negligible), and m=ρV is the mass of colloid, taken as the product between the density ($\rho_{water}=0.997\frac{g}{cm^{3}};{\;\rho }_{PEG8k}=1.0832\frac{g}{{\mathrm{cm}}^{3}}$) and the volume. The iron concentration of samples involved in hyperthermia experiments was determined using the thiocyanate assay described below. It was found that 1 mg of each type of Fe_3_O_4_ MNP contained on average 0.65 mg of iron, slightly below the theoretical value of 0.724 mg; the mass difference most probably coming from the coating of MNPs with citric acid. Thus, in the case of performing hyperthermia experiments on samples with a concentration of 1 mg_IOMNPs_/mL, the concentration of iron was 0.65 mg_Fe_/mL. To disperse the IOMNPs in PEG8K, a given mass of the polymer was heated up to 80 °C to become liquid. Then, the IOMNP powder was dispersed in 0.5 mL of the liquid polymer by ultrasonication for 15 min in an ultrasonic bath kept at 80 °C, after which the samples were allowed to cool to room temperature.

### 2.3. Cell Lines

Two types of cell lines were used in our study, one cancer cell line (human pulmonary cancer cells, A549) and one normal cell line (human gingival fibroblasts, HGF). A549 cells were procured from American Type Culture Collection (ATCC, Manassas, VA, USA) and HGF cells from Cell Lines Service (CLS, Eppelheim, Germany). Both cell types were cultured in Dulbecco’s Modified Eagle Medium (DMEM, Gibco, Paisley, UK) supplemented with 10% Fetal Bovine Serum (FBS, Sigma Aldrich, Steinheim, Germany). Cells were routinely cultured in flasks at 37 °C in a humidified incubator with 5% CO_2_ supplementation, while the medium was changed every 2–3 days. The cells were used for the experiments or subcultured once they reached 70–80% confluence.

### 2.4. In Vitro Cytotoxicity Assays

Two complementary assays were performed to assess the cytotoxicity of IOMNPs upon their incubation in cell lines for 24 h. A number of 10,000 cells for A549 and 7500 cells for HGF were seeded for 24 h in 96-well plates and incubated for 24 h to reach 70–80% confluency. The cells were exposed to 200 µL of cell medium containing IOMNPs in different concentrations: 50, 100, 200, 400, 600, 800, and 1000 µg_IOMNPs_/mL. After incubation, the cells were washed thoroughly three times with PBS (Gibco, Paisley, UK) and incubated with the Alamar Blue (AB) or Neutral Red (NR) dye. The metabolic capacity of viable cells was evaluated by the AB assay, which measures the conversion of resazurin to the highly fluorescent resorufin, a compound that permeates in the cellular media [42]. The fluorescence of resorufin in cellular supernatants was measured after an incubation of 3 h with a resazurin solution of 200 µM at λ_excitation_ = 530/25 nm; λ_emission_ = 590/35 nm using a Synergy 2 Multi-Mode Microplate Reader. The NR assay was used to measure the ATP content of exposed cells, a surrogate for cellular viability [43]. Complementary, the ATP content of cells was measured using the NR assay, a eurhodin dye that accumulates, depending on the ATP content, in the lysosomes of viable cells. Cells were incubated with an NR solution (40 μg/mL) for 2 h and subsequently washed with PBS to remove excess dye. The intracellular accumulated dye was extracted using an acidic hydroalcoholic solution (50% ethanol, 49% water, and 1% glacial acetic acid), and the fluorescence was measured at λ_excitation_ = 530/25 nm; λ_emission_ = 620/40 nm, using a Synergy 2 Multi-Mode Microplate Reader. For each condition, three biological replicates, each including six technical replicates, were performed. The results were expressed as relative to the negative control (cells exposed only to culture media).

### 2.5. Optical and Biochemical Interferences Assessment

Before the evaluation of the IOMNPs’ cytotoxicity, their optical and biochemical interferences with the viability assays were evaluated. In the case of Alamar Blue (AB), the ability of the IOMNPs to reduce resazurin to the measured fluorescent compound, resorufin, or to adsorb or re-oxidize resorufin to resazurin was evaluated by incubating different concentrations of IOMNPs with resazurin (Sigma-Aldrich, Steinheim, Germany) or resorufin (prepared *extempore* by autoclaving a resazurin solution) for 3 h. After the incubation period, the suspension mixture was centrifuged to remove IOMNPs, and the fluorescent signal of the supernatant was measured using a Synergy 2 Multi-Mode Microplate Reader (BioTek^®^ Instruments Inc., Winooski, VT, USA). Similarly, the ability of IOMNPs to interfere with the Neutral Red (NR) assay, by adsorption of the dye, was evaluated. Optical interference assays were conducted by measuring the emitted fluorescence of resorufin and neutral dye solution in an extempore prepared mixture with different concentrations of IOMNPs. IOMNPs interfered optically with both assays by quenching the emitted fluorescence (Supplementary Materials Figure S1). The interference was avoided by the measurement of the supernatant fluorescence after a centrifugation step.

Regarding the biochemical interferences, no interferences by reduction/oxidation or adsorption of the dyes were present (Figure S2). The interference of the nanomaterials with the biochemical assays used to evaluate viability is a well-known artifact that can lead to erroneous results [44]. Depending on the method of evaluation, in the case of biochemical assays based on the absorbance measurement, the presence of IOMNPs overestimates the viability due to their broad and unspecific light absorption. Conversely, in the case of fluorescent probes, an overestimation of toxicity is generally present, as the physical presence of IOMNPs quenches the emitted fluorescence. Nevertheless, the interferences can be circumvented by a proper selection of controls or by additional steps during which the nanomaterials are removed [44]. Similar to our results described hereby, Könczöl et al. reported that three types of IOMNPs with different sizes interfered only optically with the NR assay [45]. Moreover, the NR dye did not adsorb onto the IOMNPs [45].

### 2.6. Iron Concentration Determination

The iron content of samples was determined using the thiocyanate assay [46]. Briefly, the IOMNPs from 0.5 mL of a colloidal solution of acid citric coated IOMNPs (c = 4 mg_IOMNPs_/mL) were magnetically separated and further suspended in 10 mL of HCl 12% solution for digestion at 60 °C for 4 h. After the incubation, the mixture was centrifuged at 12,000 g for 10 min and the supernatants were collected. On 96-well plates, 50 µL of the supernatant was incubated for 30 min with 50 µL of 1% ammonium persulfate to oxidize all iron content to Fe^3+^. Following this step, 100 µL of 0.1 M potassium thiocyanate was added to form the colored iron-thiocyanate compound and the absorbance was measured at λ = 490 nm using the Synergy 2 Multi-Mode Microplate Reader. The iron content of IOMNPs was calculated from a Fe^3+^ standard curve with concentrations ranging between 7.8–250 µg/mL (Figure S3).

### 2.7. Evaluation of Cellular Uptake

The cellular uptake of IOMNPs was qualitatively evaluated by using Prussian blue staining and quantitatively by the Liebig reaction of free Fe^3+^ with thiocyanate in acidic medium, as described above [46]. For both assays, A549 and HGF cells were seeded in 6-well plates at a density of 3 × 10^5^ and 2.5 × 10^5^ cells per 2 mL of medium for 24 h. Subsequently, the cells were further exposed to 2 mL of IOMNPs at a concentration of 1000, 500, 250, 100, 50, and 0 µg/mL for another 24 h. After that, the cells were washed twice with PBS and once with trypsin (0.05%) to remove the IOMNPs attached to the cellular surface. For the quantitative measurement of the intracellular iron using the Liebig reaction, cells were trypsinized and centrifuged at 4500 g for 5 min, then further processed as described in Section 2.6. For Prussian blue staining, cells were fixed with 4% paraformaldehyde for 30 min at room temperature. The intracellular iron content was subsequently stained with a mixture of a 2% HCl aqueous solution and a 2% potassium ferrocyanide (II) trihydrate aqueous solution for 30 min at 37 °C. After the incubation period, cells were washed three times with double distilled water and visualized under a light microscope.

### 2.8. In Vitro Magnetic Hyperthermia

A549 and HGF cells were seeded and exposed to the same concentration of IOMNPs as described in Section 2.7. Upon the washing and trypsinization steps, the cells were dispersed in 1 mL of cell culture media. Two equal aliquots of 400 µL each were separated. The cells were gently centrifuged for 10 min at 100 g and then 200 µL of cell culture media was removed from each aliquot. Therefore, one aliquot (IOMNP loaded cells dispersed in 200 µL of cell culture) was exposed for 30 min to an Alternating Magnetic Field (AMF), while the other aliquot was immersed in a water bath maintained at 37 °C during the MH experiments to serve as a control. Three different intensities of the AMF (30, 45, and 60 kA/m) were evaluated. After the exposure to the AMF, cells were seeded in 96-well plates and the cell mortality was evaluated after 24 h using both AB and NR assays. Cell mortality was calculated as the relative ratio between cell loaded IOMNPs exposed to AMF and the control. The experiments were performed with six biological replicates, each including 3–6 technical replicates.

## 3. Results and Discussion

### 3.1. Morphological Properties

The polyol route becomes a versatile way for the synthesis of magnetic nanoparticles (MagNPs) with variable sizes, shapes, and compositions that can be easily tuned by the nature of the magnetic precursors and precipitator, the choice of the solvents, the presence of extra stabilizer, and the temperature and duration of the reaction [21,22,25,26,27,28,29,30,31,32,33,34,35,36,37,38,39,40]. In our approach, we used a constant volume of PEG200 (40 mL) as a solvent and a fixed amount of FeCl_3_·6H_2_O (1 mmol) as a magnetic precursor. It has to be mentioned that the formation of MagNPs is facilitated by an alkaline environment, which modifies the reduction potential of the polyol, being thus able to reduce the iron reactants [47]. Since the concentration of sodium acetate (NaAc) plays an important role in defining the core size and morphology of the resulting MagNPs, displaying different magnetic behavior [38], it was chosen as the precipitator in our study. Thus, the amount of NaAc was systematically varied between 0 g and 4.8 g in the reaction mixture, while its role in the growth of MagNPs was evaluated. No extra stabilizer was introduced in the synthesis protocol as the high temperature used (300 °C) might degrade it. However, the resulting MagNPs were immediately coated upon synthesis with citric acid following the protocol developed by Gavilan et al. [38]. According to this approach, the surface charge of IOMNPs is increased at neutral pH value, thus enhancing their colloidal stability through electrostatic repulsions [38]. The novelty of our approach consists of performing the synthesis at elevated temperature (300 °C), well above the boiling point of PEG200 (240 °C), which shortens the reaction time necessary to give rise to MagNPs to only 1 h.

**Figure 1:**
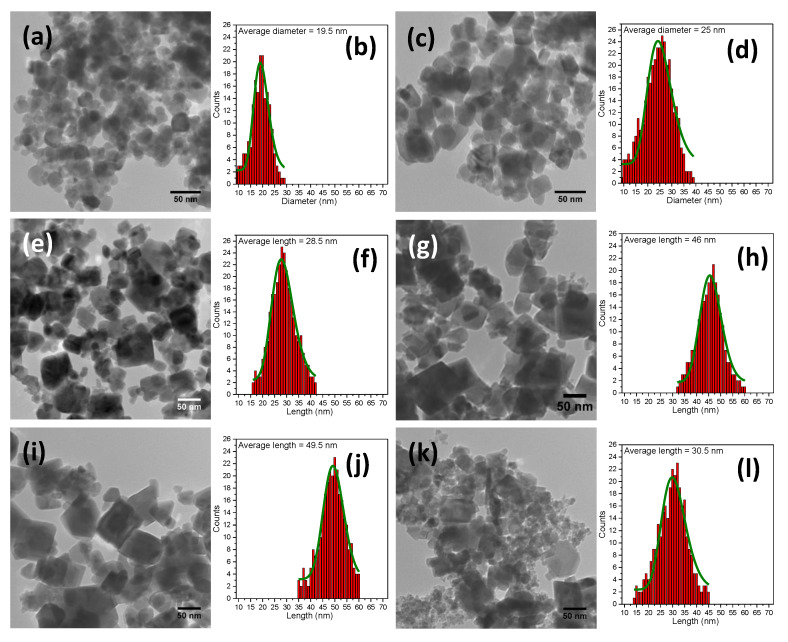
Large scale TEM images of MagNPs and their corresponding size distribution histograms fitted to a log-normal distribution (green lines) obtained by employing different amounts of NaAc in the synthesis: (**a**,**b**) 0.6 g; (**c**,**d**) 0.9; (**e**,**f**) 1.2 g; (**g**,**h**) 2.4 g; (**i**,**j**) 3.2 g; and (**k**,**l**) 4.8 g.

Despite working at elevated temperature, in the absence of NaAc in the reaction mixture, no formation of MagNPs occurred, as previously reported [30,48]. The use of a small amount of NaAc (0.1 g) in the reaction mixture led to the formation of small grains (a few nanometers in diameter) assembled in spherical structures with a diameter of several hundreds of nanometers (Figure S4a). By increasing the NaAc amount to 0.3 g, the grains constituting the big spherical structures increased in diameter (Figure S4b). With the use of 0.6 g of NaAc in the reaction mixture, individual spherical MagNPs can be clearly distinguished (Figure 1a).

The log-normal size distribution, obtained by manual measurement of the diameter of around 300 MagNPs through ImageJ software and further data fitted using Origin software, indicated an average diameter of 19.5 nm for the sample (denoted MagNP1) (Figure 1b). This value increased to 25 nm when 0.9 g of NaAc was employed in the reaction mixture (Figure 1c,d). Some MagNPs with irregular shapes could be also observed among the spherical MagNPs in the sample denoted MagNP2 (Figure 1c). Polydispersed polyhedral MagNPs with an average length of 28.5 nm (denoted MagNP3) started to form once 1.2 g of NaAc was introduced in the reaction mixture (Figure 1e,f). By doubling the amount of NaAc (2.4 g) in the synthesis (MagNP4), the polyhedral shape of MagNPs was clearly defined and became more frequent, whereas the average length increased to 46 nm (Figure 1g,h). The MagNPs kept the polyhedral form (MagNP5) and slightly increased its average length to 49.5 nm by further increasing the amount of NaAc at 3.6 g in the reaction mixture (Figure 1i,j). Finally, a mixture of polyhedral and irregular shaped MagNPs with an average length of 30.5 nm (MagNP6), was formed when the amount of NaAc was increased to 4.8 g (Figure 1k,l). Possibly due to the drying process on the TEM grid, the MagNPs appeared under the TEM images as aggregated in all samples, but no specific assembly of the MagNP was observed (Figure 1).

### 3.2. Structural Properties

**Figure 2:**
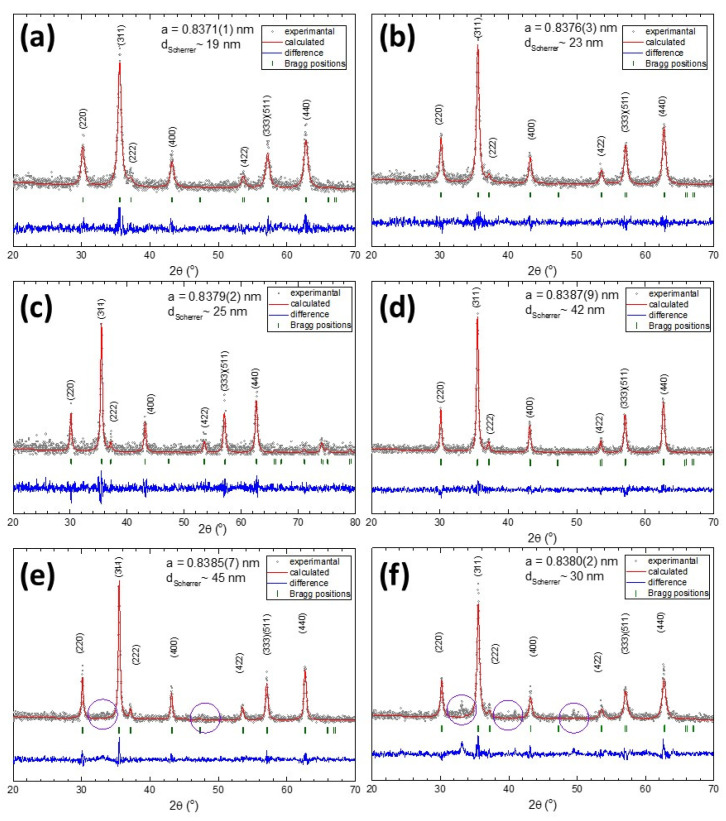
XRD diffractions patterns of MagNPs obtained by employing different amounts of NaAc in the synthesis: (**a**) 0.6 g, (**b**) 0.9, (**c**) 1.2 g, (**d**) 2.4 g, (**e**) 3.2 g, and (**f**) 4.8 g. The average diameters calculated based on the Scherrer equation and the lattice parameters are also indicated for each type of MagNP. The purple circles highlight the diffraction peaks probably arising from the excess of NaAc used in the reaction mixture.

The XRD patterns, presented in Figure 2, clearly revealed the existence of a pure inverse spinel crystalline structure in all six samples. The position and the relative intensities of all diffraction peaks were ascribed to magnetite Fe_3_O_4_ (PDF number: 88-0315 [49]). Peaks related to other crystalline phases like FeO or Fe_2_O_3_ were not found in the XRD patterns, indicating that all MagNPs consisted of pure magnetite Fe_3_O_4_. Instead, in the diffractograms of both MagNP5 and MgNP6 samples, unidentified tiny peaks appeared (purple circles in Figure 2e,f), probably due to a crystalline structure of a different nature than the magnetic one. These peaks were more visible in the diffractogram of the MagNP6 sample (Figure 2f) synthesized with 4.8 g of NaAc, suggesting the idea that they may arise from the excess of NaAc used in the reaction that crystallizes onto the surface of MagNPs.

A progressive decrease in the width of the diffraction peaks, indicating an increase in the crystallite size, was observed when going from MagNP1 to MagNP5. The crystalline size of all MagNPs, calculated using Scherrer’s equation by Gaussian fit of the (220), (311), and (440) peaks, agreed well with the average length obtained from the TEM images (Table 1). Since the sizes revealed by the XRD data corresponded with the smallest crystallites, which are responsible for the largest widths in the XRD peaks, the relatively broad size distribution of all six types of IOMNPs suggests that most of them are single crystals. The corresponding lattice parameters of MagNPs, listed in Table 1, were very close to that of bulk magnetite (a = 0.8375 nm). From TEM and XRD data, it is clear that increasing the amount of NaAc in the reaction mixture up to 3.6 g leads to MagNPs with larger core sizes.

### 3.3. Nanoparticle Magnetism

**Figure 3:**
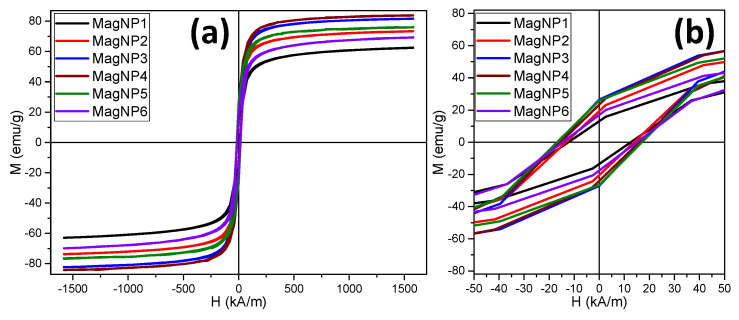
(**a**) Magnetization curves and (**b**) their low-field regime for all types of MagNPs recorded at 300 K.

The magnetic hysteresis (M–H) loops of the as-prepared Fe_3_O_4_ MagNPs were collected at room temperature (Figure 3a). In the low-field region, hysteresis loops existed (Figure 3b) for all six types of MagNPs, indicating ferromagnetic behavior at room temperature with remanence and coercivity. The values of the ratios M_r_/M_s_ were smaller than 0.5 for all samples, indicating a uniaxial anisotropy. Therefore, the anisotropy constants were calculated as K_eff_ = μ_0_ H_c_ M_s_/0.96 [50]. As depicted in Table 2, the samples MagNP1 and MagNP2, formed by spherical IOMNPs, exhibited the lowest values of K_eff_, M_s_, H_c_, and M_r_. The values of these magnetic parameters increased for the next three samples: MagNP4, MagNP5, and MagNP6, which represent polyhedral IOMNPs (Table 2). The larger sizes and the polyhedral shape, which lowers the spin canting and the number of surface disorder spins [51], of these type of MNPs considerably increase their M_s_. On the other hand, the polyhedral MNPs displayed an increased anisotropic shape compared to spherical MNPs (MagNP1 and MagNP2), which also contributed to the increase in both H_c_ and M_s_, as in the case of nanorods [52]. For the last sample (MagNP7), which was composed of a mixture of spherical and polyhedral MagNPs, the three magnetic parameters decreased to values situated in between those recorded for spherical and polyhedral MagNPs (Table 2).

From Table 2, it can be observed that the polyhedral MagNPs from sample MagNP3 and MagNP4 exhibited the highest values of M_s_ of 80 and 83 emu/g, respectively. These high values of M_s_ were very close to the reported value of 84 emu/g for the commercial magnetite powder and that of the magnetite nanoparticles synthesized by high-temperature thermal decomposition of iron organic salts [53]. Compared to the polyhedral IOMNPs synthesized at 240 °C for 6 h [33], the increase of the reaction temperature to 300 °C for 1 h considerably improved the crystallinity of the IOMNPs, leading to a value of M_s_ approaching the bulk value as well as an increase of the H_c_ by 10 kA/m.

### 3.4. Hyperthermia Properties

**Figure 4:**
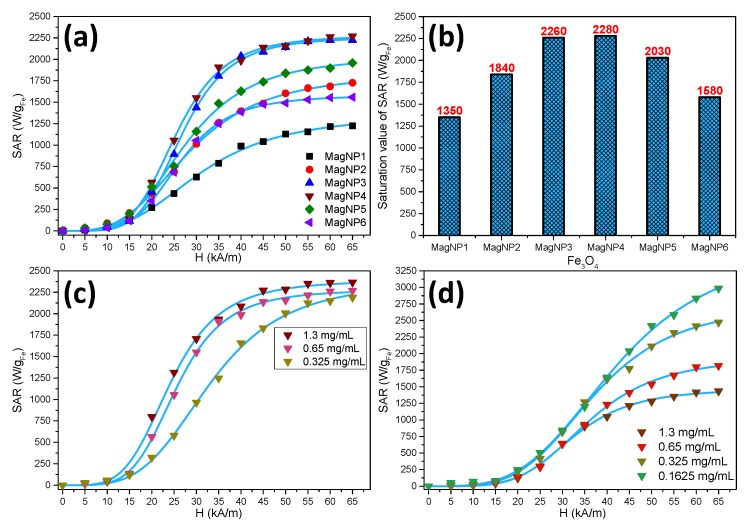
(**a**) Specific Absorption Rate (SAR) dependence on H for all six types of MNPs dispersed in water at an iron content of 0.65 mg/mL. (**b**) The value of SAR_max_ for all six types of MNPs; SAR dependence on H for MagNP4 dispersed in (**c**) water and (**d**) solid PEG8K at different iron contents. Blue lines represent the fits with the logistic function.

To get a clear picture of the heating efficiency of the six types of IOMNPs, their SAR was evaluated in water at the same concentration. The heating curves of MNPs fitted with the Box–Lucas equation are presented in Figure S5. For all six types of IOMNPs, the AC magnetic field amplitude (H) dependence of SAR (Figure 4a) can be divided into three regions. In the first region, defined by low values of H (0–15 kA/m), the SAR values were insignificant since the hysteresis area was very small. Once the H_c_ of MNPs (12–17 kA/m, Table 2) was exceeded by a higher value of H (20 kA/m), the hysteresis area was larger, and the IOMNPs started to deliver heat. Therefore, in the second region, defined by H values between 15 and 45 kA/m, the SAR values of IOMNPs increased abruptly up to the saturation values. The plateaus of SAR values defined the third region in Figure 4a for H values higher than 45 kA/m.

As described in our previous papers [32,33,54], the evolution of SAR values with H, in the case of ferromagnetic MNPs, presented a sigmoidal shape qualitatively in perfect agreement with the numerical simulation performed by Carrey et al. [55] and Mehdaoui et al. [56], based on Stoner and Wohlfarth’s model derived theories [50]. The sigmoidal evolution of SAR with H can be well fitted (R^2^ > 0.999) phenomenologically with a simple logistic function:

(3) $$SAR=SAR_{MAX}\frac{{\left(\frac{H}{H_{cHyp}}\right)}^{n}*\propto }{1+{\left(\frac{H}{H_{cHyp}}\right)}^{n}*\propto }$$

with:

(4) $$\propto =\frac{n+1}{n-1}$$

where SAR_MAX_ represents the saturation value of the SAR; H_cHyp_ is the hyperthermic coercive field (not identical with the coercive field H_c_ obtained in DC magnetic measurements); the value of H for which the function presents the higher slope [55]; and the exponent n indicates how steep is the dependence of SAR on H (blue curves in Figure 4a).

As observed from Table 3, the hyperthermia coercive field (H_cHyp_), which also represents the magnetic field at which the first derivative of SAR against the H presents a maximum [33], does not vary significantly for all six types of MNPs in accordance with the slight variation of their H_c_, measured in DC conditions (Table 2). Instead, the exponent n, which indicates how steep the dependence of SAR is on H, increased from 3.24 for spherical MagNP1 to 4.85 for polyhedral MagNP4 (Table 3), suggesting that the heating behavior of these type of MNPs was closer to an ideal Stoner Wohlfarth model [50]. The most important variation was recorded for SAR_max_, which represents the saturation value of the SAR. As depicted in Figure 4b and Table 3, among all six samples, the polyhedral MNPs from samples MagNP3 and MagNP4 exhibited the highest value of SAR_MAX_, reaching 2260 W/g_Fe_ and 2280 W/g_Fe_ for an iron content of 0.65 mg/mL, respectively. As the values of H_c_ of all six types of MNPs did not vary significantly (Table 2), it can be considered that the important variation of the SAR_MAX_ (Figure 4b) is mainly related to the variation in M_s_. As shown in Figure S6, there was an almost linear dependence between the SAR_MAX_ values and M_s_ among these samples possessing very similar H_c_. The polyhedral IOMNPs belonging to the sample MagNP4, exhibiting the best magnetic hyperthermia properties, were selected for further investigations.

When the concentration of the IOMNPs in water was decreased from 1.3 mg_Fe_/mL to 0.65 mg_Fe_/mL and further to 0.325 mg_Fe_/mL (Figure S7), we calculated a monotonous change of the H_cHyp_, which increased from 21.71 kA/m to 28.98 kA/m while the SAR_MAX_ varied slightly between 2280 W/g_Fe_ and 2390 W/g_Fe_ (Figure 4c and Table 3). One can notice a decrease in the exponent n from 4.93 to 3.90 when the concentration of IOMNPs decreased from 1.3 mg/mL to 0.325 mg/mL. It is quite obvious that for biological applications, one must consider the evaluation of the hyperthermia properties of IOMNPs in an environment close to the in vivo and in vitro characteristics. Taking into account that the IOMNPs are internalized by the cells in endosomes [33], which facilitates their aggregations, thus considerably reducing their mobility, we performed SAR measurements for the IOMNPs dispersed randomly and uniformly in a solid matrix (PEG8k) at different concentrations (Figure S8). The SAR kept a similar sigmoidal shape evolution with H (Figure 4d). For all the samples measured in PEG8k, we measured a dramatic increase in the values of H_chyp_, ranging from 29.51 kA/m to 35.76 kA, with the highest values at the lowest concentration, accompanied by a significant increase in the SAR_MAX_ values (up to 3620 W/g_Fe_ at the lowest concentration of 0.1625 mg_Fe_/mL). A similar decrease was recorded for the exponent n as the concentration of IOMNPs decreased.

As one can notice, there was a striking difference in the behavior of SAR evolution with H as a function of the concentration of IOMNPs in the two different media. In the low field regime, the SAR increased with increasing concentration of IOMNPs in water, while in PEG8k, the SAR decreased with an increase in the concentration of IOMNPs. In water, close to saturation, similar values of SAR were obtained at the tested concentration, while in PEG8k, the low-field behavior was preserved (i.e., SAR decreased with increasing concentration). Our previous data indicated a similar type of SAR dependence on the concentration of MNPs and H for both manganese and zinc ferrites, in water and PEG8k [54]. Moreover, we demonstrated that the alignment of the MNPs in and the external DC field at high temperature in liquid PEG8k, before solidification, led to a decrease in H_chyp_ and an increase of the SAR_MAX_ values. Based on the fact that the decrease in H_chyp_ occurred only in water where the MNPs were mobile, a similar effect was observed in MNPs pre-aligned in DC magnetic fields in solid PEG8k, which can be explained by the formation of chains of MNPs in water under the action of AMF [54]. We consider that this explanation is also valid for the IOMNPs presented in this study, since H_chyp_ decreased in both situations, either when passing from PEG8K to water, or when increasing the MNP concentration. Both the dispersion of MNPs in an environment like water (where they keep their mobility) and the increase of their concentration favor the chain formation under the effect of the AMF. This behavior is typically observed for highly coercive ferromagnetic IOMNPs, as shown in the case of nanorods [57] or 35 nm magnetite nanoparticles suspended in water, as suggested by AC hysteresis, susceptibility, and SAR data [58]. Other reports have also shown that the changes in magnetic susceptibility data (Ms, Mr, χ) are related to the length of the chains formed by the MNPs [59]. Moreover, it has also been reported that while MNPs organized in chains displayed a highly increased anisotropy, this is related not to their intrinsic properties but their collective behavior, more specifically, to the demagnetizing factor of the chain [60]. The influence of the chain formation on the squareness of the AC hysteresis loops was also reflected in our samples by the value of the exponent n; the higher the n value, the closer the hysteresis loop is to the ideal square shape of a pure Stoner-Wohlfarth one. As one can observe (Table 3), the n values increased as the concentration increased and higher concentrated colloids were more prone to form chains. Moreover, we obtained higher n values in water compared to PEG8k, once again, the water being an environment allowing the chain formation. On the other hand, the significant increase in the SAR_MAX_ values with decreasing concentration (leading to an increase in the mean distance between the nanoparticles), which was more obvious in the case of PEG8k immobilized IOMNPs, can be explained by the decrease in their dipolar interactions, as generally accepted in the literature.

Another interesting feature revealed by our experimental data is related to the differences in the SAR_MAX_ values between the samples measured in water and those measured in PEG8k. At higher concentrations (1.3–0.625 mg_Fe_/mL), the immobilization of the IOMNPs in the solid matrix led to a decrease in their hyperthermia performance compared with the behavior in water (Figure S6a,b). This effect is usually explained by the blocking of the physical rotation of the nanoparticles and subsequently of their Brown relaxation mechanisms. For IOMNPs with sizes larger than 20 nm, as in our case, Brown relaxation was the main relaxation mechanism, the Neel relaxation mechanism prevailing for smaller size ranges. Thus, as heat dissipation involves both hysteresis and Brown heating, blocking the latter reduces the overall heat dissipated by the IOMNPs. However, in the case of more diluted samples (0.325–0.1625 mg_Fe_/mL), higher SAR_MAX_ values in PEG8k were obtained compared to water (Figure S9c). We believe that these differences might be explained by a better dispersion of IOMNPs in PEG8k. To measure their heating properties in PEG8k, the IOMNPs were first dispersed in liquid PEG8k at 80 °C, ultrasonicated for 15 min in a water bath kept at 80 °C, and then allowed to cool to room temperature. This procedure also produced a better dispersion of the IOMNPs because at the higher temperatures reached in this process, the remnant magnetization was reduced and therefore, the interactions between individual nanoparticles were reduced. This individual organization of the suspension is preserved when the sample is solidified. While for the aqueous samples we also proceeded to 1 min sonication before each SAR measurement, one cannot exclude small associations between neighboring particles due to their nonzero remanence. An alternative experimental approach reported recently [61] is to introduce the MNPs in submicrocavities, preventing their aggregations in the cellular environment and preserving their heating capabilities.

### 3.5. Cytocompatibility of Magnetite Nanoparticles (IOMNPs)

**Figure 5:**
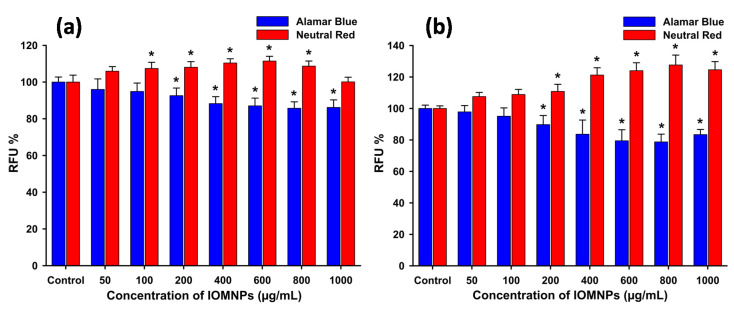
Cytocompatibility of magnetite nanoparticles (IOMNPs) on (**a**) Human Gingival Fibroblast (HGF) and (**b**) human cancer pulmonary (A549) cell lines, evaluated after 24 h exposure. Cellular viability was measured using two complementary assays, namely Alamar Blue and Neutral Red. The values are expressed as mean ± SD of six biological replicates. Data are expressed as relative values to the negative control. Asterisks (*) indicate significant differences compared to the negative control (ANOVA + Dunn’s; *P* < 0.05).

The polyhedral IOMNPs displayed high biocompatibility over the tested dose range on both cell types, while no significant differences between the two cell types were observed (Figure 5a,b). In the case of the AB assay, a statistical decrease in the viability of up to ≈ 20% was present, starting from the dose of 200 µg_IOMNPs_/mL, for both types of cells. It is generally conceded that a survival cell rate superior or equal to 80% indicates a non-toxic effect [62]. The results obtained are in agreement with our previous results based on MTT assay, where exposure of normal and cancerous cells to small (≈34 nm) and large (≈270 nm) IOMNPs at a maximal dose of 200 µg/mL resulted in a mild decrease in cellular viability, not exceeding 20% [33]. The high biocompatibility of IOMNPs is advantageous in the clinical settings, as is the case of ferumoxytol, an IOMNP approved by the FDA for the treatment of iron-deficiency anemia in adults with chronic kidney disease [63]. Similar to our findings, ferumoxytol was shown not to induce cellular death at doses up to 1200 μg/mL in mammary carcinoma cells [63]. As compared to AB assay, where a slightly dose-dependent decrease in the viability was recorded, in the case of NR assay the viability of both cell types apparently increased with increasing the IOMNPs dose. The different behaviors of the cellular viability, observed between the two complementary assays, are not due to interferences. Since NR assay is based on the ATP-dependent lysosomal incorporation of the supravital dye and the measurement of the fluorescence of the incorporated dye [43], the apparent increase in the viability given by NR assay is most probably the result of an increased lysosomal compartment due to the IOMNPs exposure and intra-lysosomal incorporation. Dissimilarities between viability assays that evaluate different mechanisms of toxicity were previously reported, reiterating the need for multiple viability assays when evaluating the cytocompatibility/cytotoxicity of nanomaterials [64,65]. Similar to the results obtained in this study, a slight decrease in the cellular viability, measured by the WST assay—an assay similar to Alamar Blue-, and an increase in the NR-dye uptake upon exposure to IOMNPs were previously reported [44]. Thus, the increased uptake of NR-dye might be correlated to the accumulation of IOMNPs in lysosomes. The higher sensitivity of either WST or Alamar Blue viability assay could be related to mitochondrial damage by IOMNPs, which results in a decreased conversion of formazan or resazurin by mitochondrial diaphorases. Moreover, the mitochondrial impairment of nanomaterials is presumed to be related to their redox-active surface that hinders the electron flow and the mitochondrial functionality [66].

### 3.6. Cellular Uptake of IOMNPs

**Figure 6:**
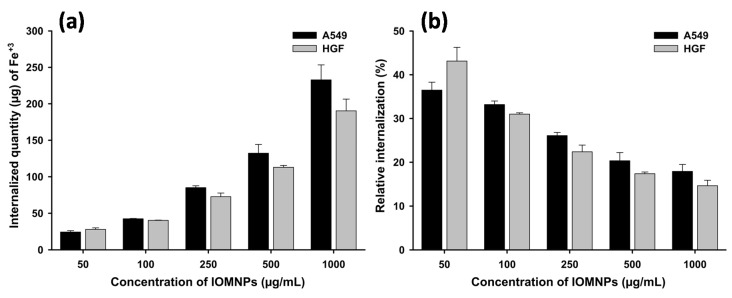
The amount of Fe^+3^ internalized in HGF and A549 (**a**) and the relative internalization (**b**) evaluated after 24 h of exposure to different concentrations of IOMNPs. The values are expressed as mean ± SD of at least three biological replicates.

The cellular internalization of IOMNPs in HGF and A549 cells was evaluated both qualitatively and quantitatively by staining the intracellular iron content with Prussian blue and by quantifying it, based on the reaction of digestion-free ferric ions and thiocyanate [46]. The cells were exposed for 24 h to different concentrations of IOMNPs (1000, 500, 250, 100, and 50 µg_IOMNPs_/mL) and subsequently washed thoroughly with PBS and then trypsin, to discard superficially attached IOMNPs. As depicted in Figure 6a, the amount of iron internalized in both cells increased non-linearly as the dose of IOMNPs increased (see Table S1). In contrast, the relative internalization of IOMNPs (ratio between the internalized IOMNP amount and the exposed IOMNP amount) decreased as the dose of IOMNPs increased (Figure 6b and Table S1), indicating that the IOMNPs are mainly internalized through an active transport system. Endocytosis, an active transport process, relying on the invagination of the plasma membrane at clathrin-coated pits or caveolae, is considered as the dominant process involved in nanoparticle cellular uptake [67]. These findings are rather different from the results reported by Matsuda et al., where the amount of IOMNPs incorporated into three different mesothelioma cells increased linearly with the dose of IOMNPs [68]. We speculate that the last washing step with trypsin performed in our study detached an additional quantity of superficially bounded IOMNPs that would otherwise induce a linear dependence of internalized amounts of IOMNPs on their dose. Aside from the lowest dose of 50 µg/mL, a statistically higher amount of iron was observed to be internalized in the cancerous A549 cells compared with the normal HGF cells. The higher incorporation of IOMNPs in A549 cells is in agreement with the current data, cancerous cells displaying, in general, a higher internalization than normal cell types [69,70,71,72]. Due to their increased metabolic activity and division rate, which require an increased demand for nutrients, cancerous cells have a higher endocytotic potential and a higher nanoparticle uptake [73]. Statistical analysis (Two-way ANOVA + Holm-Sidak) revealed that the dose variable (*p* < 0.001) had a greater influence than the cellular type (*p* = 0.024) on the IOMNPs’ cellular internalization.

**Figure 7:**
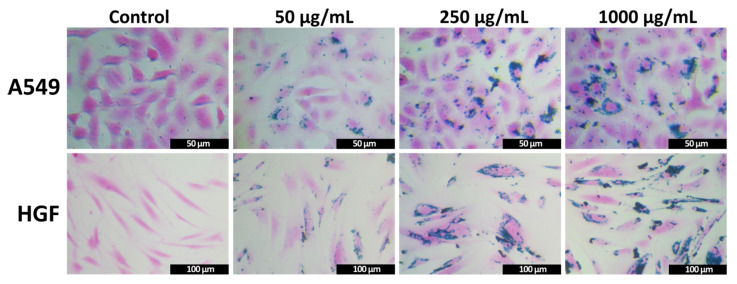
Microscopic images of A549 (upper panels) and HGF (lower panels) cells incubated with Prussian blue stained IOMNPs at different concentrations 0, 50, 250, and 1000 μg_IOMNPs_/mL for 24 h.

Aside from the amount of IOMNPs internalized by cells, microscopic images of both types of cells upon 24 h incubation with Prussian blue stained IOMNPs revealed the final location of IOMNPs within the intracellular compartment. As can be observed in Figure 7, the amount of internalized IOMNPs increased with an increase in the exposure dose within the cytoplasm, but not in the nucleus of the A549 and HGF cells. Independent of the exposure dose, the cells presented a normal morphology with no shrinkage of the cellular volume, indicative of a cytotoxic effect (Figure 7). The size, outer surface structure, and adherence were not negatively influenced by the exposure to the IOMNPs. The washing step with trypsin appeared to be constructive as it has removed most of the superficially-bound IOMNPs from the cellular membranes. Similar to other studies, our IOMNPs were not internalized uniformly by the cells, some cells displayed a cytoplasmatic overload, while others contained a lower amount of IOMNPs [74,75,76]. Consequently, the exposure of cells to AFM might result in a different response, depending on the quantity of IOMNPs internalized by each cell, as previously demonstrated by Catalayud et al. [76].

### 3.7. In Vitro Magnetic Hyperthermia

**Figure 8:**
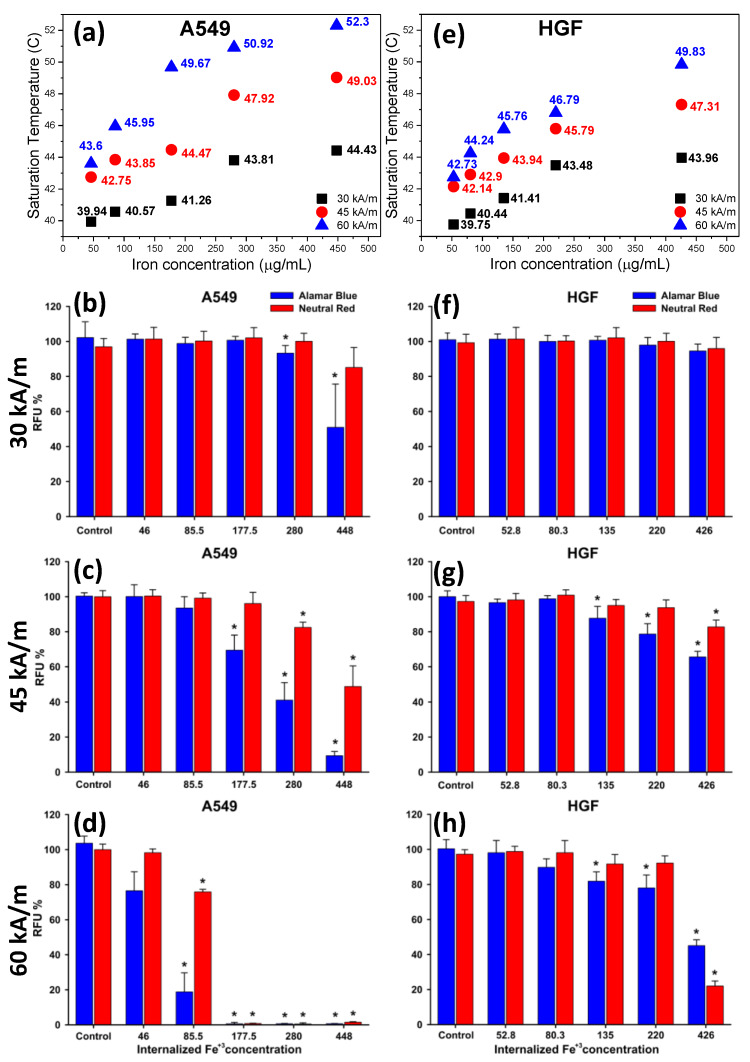
Saturation temperatures and cytotoxic effect of internalized IOMNPs on (**a**–**d**) A549 (left panels) and (**e**–**h**) HGF (right panels) evaluated after a 30 min exposure to three different values of H (30, 45, and 60 kA/m). Cellular viability was measured using Alamar Blue and Neutral Red assays. The values are expressed as mean ± SD of three biological replicates. Data are expressed as relative values to the negative control. Asterisks (*) indicate significant differences compared to the negative control (ANOVA + Dunn’s; *P* < 0.05).

Before the evaluation of the MH efficiency in selectively killing cancerous cells, the influence of the AMF on cell integrity was evaluated for both cell types. No significant decrease in cellular viability was observed upon a 30 min exposure of both cell types to AMF, as reported by several research groups [72,74,77,78]. The recorded temperatures during these tests were close to 38 °C. In contrast, an increase in the recorded temperatures was observed when the IOMNP incubated cells were exposed to AMF (Figure S10). The saturation temperature (when the heat released by internalized IOMNPs equals the dissipated heat into the environment) depends on H, the amount of internalized IOMNPs, and the evaluated cell type (Figure 8a,e, and Figure S10). Statistical analysis based on the three-way ANOVA, considering as variables the cell type, amount of internalized IOMNPs, and H showed that all three variables significantly influenced the measured viabilities (AB and NR assays) with individual *p* values smaller than 0.001.

At the lowest H (30 kA/m), no cell death was observed in the case of HGF cells over the entire dose range as evaluated with both toxicity assays (Figure 8f). Instead, a statistical decrease in the A549 cell viability, based only on the AB assay, was observed at the highest two doses (Figure 8b). This is in agreement with the slightly higher increase in temperature, noticed in the case of A549; the saturation temperature reaching values with 0.5 °C higher than those recorded on HGF cells (Figure 8a,e). The difference in the saturation temperatures could be due to the different amount of IOMNPs internalized in both cells, which was more obvious at the highest exposed doses (Figure 6a). Please note that the new doses in Figure 8 were evaluated based on the internalized amount of IOMNPs inside both cell types and the in-vitro MH experimental methodology (Table S1). Additionally, the decrease in the A549 viability could also be explained by the fact that cancer cells are much more sensitive to temperatures between 42–45 °C than normal cells [79]. Similar to the observation made in the biocompatibility evaluation experiments, the AB assay was more sensitive than the NR assay. By increasing H to 45 kA/m, the saturation temperatures increased over the entire dose range for both types of cells (Figure 8a,e). For the highest two doses, the saturation temperatures reached 48–49 °C in the case of A549 cells (Figure 8a) with 2 °C higher than for HGF cells (Figure 8e). Thus, the cytotoxicity was more pronounced in A549 cells, as indicated by both assays (Figure 8c,g). Based on the AB data, the cellular viability for A549 decreased below 50% (Figure 8c) compared to the HGF normal cells, which were more resilient (Figure 8g). Although the saturation temperatures obtained for the highest dose at H = 30 kA/m were close to those reached for the intermediate dose at H = 45 kA/m for both types of cells, a higher number of cells died in the latter case. At the lowest two doses, no significant cell death was observed in both cases (Figure 8c,g), which was in agreement with the low saturation temperatures reached.

The in vitro MH experiments carried out with H = 60 kA/m on IOMNPs loaded A540 cells enabled reaching saturation temperatures close or above 50 °C (Figure 8a). Independently of the biochemical assay used, the AMF exposure induced overt cytotoxicity of A549, the observed cellular viability being close to zero for the highest two doses and intermediated one (Figure 8d). Although a large discrepancy was observed between the measured viabilities (20% vs. 80%) using both assays, a statistically significant decrease in viability was observed at the next lower dose (Figure 8d). Less pronounced toxicity in comparison with the cancerous cells was recorded for normal cells (Figure 8h). At the highest exposure dose, the cellular viability measured with the AB and NR assays dropped to 45% and 20% as the saturation temperature reached almost 50 °C. For the next four doses, the increase of H to 60 kA/m did not substantially induce an additional cytotoxic effect in comparison with H = 45 kA/m (Figure 8g,h). Although the reached saturation temperatures were higher (Figure 8e), the cellular viabilities were almost similar, indicating that the normal cells could withstand temperatures of 43–46 °C, in contrast to cancer cells, which partially undergo apoptosis at those temperatures.

If we compare the viability of the cells as a function of the saturation temperatures reached for different IOMNP doses and different amplitudes of the AMF, we observed that the saturation temperature was not the main parameter dictating the rate of cell survival. In fact, one can notice that at almost the same saturation temperature reached for different doses, the viability rate was strongly influenced by the amplitude of the magnetic field, with lower viability rates at higher H values. This discrepancy was more obvious at higher field strengths.

**Figure 9:**
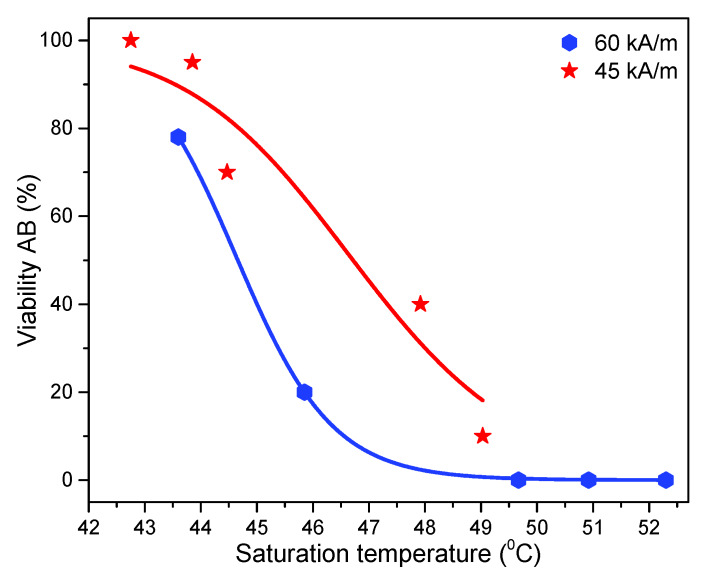
The viability data for A459 cells based on the AB assay as a function of saturation temperature for different amounts of internalized IOMNPs by the cells. The lines represent fittings with the Hill function.

As can be seen in Figure 9, where the viability rates were plotted against the saturation temperatures for two amplitudes of the AMF (45 kA/m and 60 kA/m) based on the AB assay, it is obvious that at the same saturation temperatures, the viability rates were lower at the higher field strength.

The more pronounced biological effect obtained at the same saturation temperature but with higher amplitude of the AMF might be due to the mechanical injuries of the membrane and cytoplasmatic structures induced by the mechanical actions developed by the internalized IOMNPs when subjected to the AMF [80,81], in addition to the caloric stress that results in protein denaturation. Moreover, the physical disruption of membrane structure and the increase in the membrane fluidity and permeability was hypothesized to be the mechanism of action behind the increased susceptibility of cancerous cells to different cytotoxic and cytostatic drugs after combined therapy with MH [82,83,84]. On the other hand, the temperature measured by macroscopic probes could differ significantly from the temperature in the near-vicinity of the cellular-internalized IOMNPs, where higher temperatures are expected. This temperature gradient was previously pinpointed in hyperthermia experiments carried out at similar temperatures, achieved by heating the IOMNP loaded cells either under MF or in water-bath, that demonstrated a higher cellular death in the former case [74,82,85]. The local increase of the temperature at the surface or in the proximity of IOMNPs might induce cellular death through lysosomal pathways. Creixell et al. [86] have demonstrated that Epidermal Growth Factor Receptor (EGFR) targeted MNPs were able to induce cancer cell death without measuring a macroscopic temperature change. Furthermore, Domenech et al. [14] showed that the potential mechanism in cell death was related to the accumulation of MNPs in cell lysosomes and the subsequent lysosome membrane disruption, upon the application of an AMF. Aiming at elucidating the lysosomal mechanism of cell death induced by MNPs, Clerc et al. [13] used MNPs targeting lysosomes of tumor cells to deliver magnetic intralysosomal hyperthermia (MILH). Their results suggest that cell death occurs through a local temperature increase at the surface at the MNPs, with subsequent enhancement of ROS production leading to lipid peroxidation, lysosomal membrane permeabilization, and leakage of lysosomal enzymes into the cytosol, which activates Caspase-1. Connord et al. [87] used a miniaturized electromagnet setup and an inverted confocal microscope, allowing them to follow in real-time the mechanisms of cell damage induced by IOMNPs under an AMF. Their results showed that cell damage is an event occurring at the single-cell level, the mechanisms involved being the lysosome membrane permeabilization and ROS formation during AMF treatment. Moreover, they observed the lysosome alignment in chain-like structures under the action of the AMF. Although our IOMNPs were not specifically functionalized to target lysosomes, they first accumulated in lysosomes, as suggested by our results of the increased NR, a lysosomal dye, accumulation in the cells, when the latter were exposed to IOMNP uptake experiments. This preferential lysosome accumulation of IOMNPs leads to local heating, which depends on the amplitude of the AMF and can trigger ROS production and the other mechanisms described above, finally leading to cell death.

**Figure 10:**
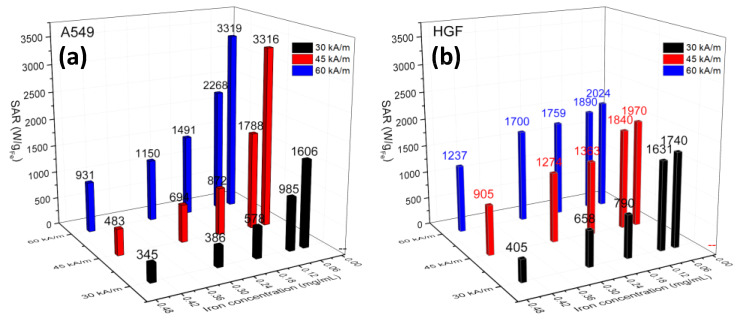
SAR dependence on the concentration of the internalized IOMNPs in (**a**) A549 and (**b**) HGF cells for each evaluating H values of 30, 45, and 60 kA/m.

The SAR values of internalized IOMNPs in both types of cells, calculated based on Box–Lucas fitting of heating curves (Figure S11), are presented in Figure 10. It can be observed that for each concentration of internalized IOMNPs, the SAR values increased as the H was varied from 30 kA/m to 45 kA/m and finally to 60 kA/m, in agreement with the increase in the saturation temperatures (Figure 8a,e). Moreover, as observed in PEG8k for the same type of IOMNPs (Figure 4d), the SAR values increased as the internalized IOMNP dose decreased for both types of cells. However, the evolution of SAR values with the internalized IOMNP dose for each H was different for the two types of cells. In the case of A549 cells, a steep increase of SAR with a decrease in the doses was recorded, while for HGF cells, the SAR values tended to saturate at the two lower doses. It might be speculated that the larger HGF cells, displaying a contact surface three times higher than A549 cells, may accommodate a higher number of IOMNPs per cell. Even though the incubation dose of IOMNPs is decreased, the number of IOMNPs within endosomes would not vary too much, producing a minute decrease in the interparticle dipole–dipole interaction and thus a slight increase in the SAR values.

On the contrary, the faster growth and a smaller contact surface of A549 cells might lead to the distribution of IOMNPs in a greater number of cells as the incubation dose of IOMNPs is decreased. Consequently, the mean interparticle distance and the dipolar interaction would decrease more pronounced in A549 cells, in this way leading to the observed steeper increase of the SAR values. Aside from this, at higher doses, the IOMNP accumulation is smaller in A549, (which might accommodate more IOMNPs), thus having a smaller interparticle distance and increased dipolar interaction, accounting for the recorded values of SAR. This scenario might explain the higher values of SAR in the case of HGF cells than those obtained for A549 cells, as clearly shown in Figure 10, particularly for the first three doses. The extremely important effects of how IOMNPs organize themselves in clusters and the sizes of these clusters were also revealed by Niculaes et al. [88], who showed that controlled grouping of nanoparticles in so-called “dimers” and “trimers” composed of two and three nanoparticles, respectively, increased SAR values, while conversely, forming centrosymmetric clusters with more than four nanoparticles led to lower SAR values. This means that slight changes in the number of IOMNPs entering in such clusters can lead to significant changes in the SAR values. As shown in Figure S12, the SAR values recorded on both types of cells were smaller than those obtained in PEG8k at almost similar IOMNPs concentrations, indicating that in the latter case, there was a better dispersion and thus a reduced interparticle interaction.

## 4. Conclusions

Increasing the synthesis temperature to 300 °C within the polyol method significantly reduces the synthesis time to 1 h (from the usual 6–12 h), leading to polyhedral IOMNPs with improved magnetic (M_s_ = 83 emu/g) and hyperthermia properties (SAR_MAX_ ~2400 W/g_Fe_ in water). The use of NaAc is essential for a successful synthesis; the optimum molar ratio between NaAc and iron salt is 18:1 for obtaining faceted IOMNPs with a reduced spin canting effect. The use of very high AMF amplitude H (up to 65 kA/m) revealed a sigmoidal dependence of SAR on H, as described by Stoner and Wohlfarth model derived theories. The concentration dependence of SAR suggests chain formation by the IOMNPs dispersed in water under the action of the AMF, as reported recently by other groups. Experiments performed with the IOMNPs uniformly dispersed in solid PEG8k showed a monotonous increase in the SAR_MAX_ values (reaching 3620 W/g_Fe_) as the concentration decreased, underscoring the extremely important role of a proper dispersion of the IOMNPs on their heating performances.

Cytotoxicity studies on two cell lines (A549 cancer cell line and HGF normal cell line) using AB and NR assays revealed that the cells internalized the IOMNPs in a dose-dependent manner, with higher amounts of IOMNPs in A549 cells. Moreover, the increase in the NR (a lysosomal dye) uptake when the cells were exposed to IOMNPs suggests that, at least partly, the IOMNPs were internalized inside the lysosomes. Intracellular hyperthermia experiments revealed that 30 min AMF exposure of the IOMNP loaded cells led to a significant increase in the macroscopic temperatures of their suspensions up to saturation temperatures of 52.3 °C and 49.8 °C for A459 cells and HGF cells, respectively. As expected, the cancer cells were more sensitive to intracellular hyperthermia compared to the normal ones. The most interesting result was that for the same saturation temperatures reached (for different IOMNPs loadings), cells exposed to higher amplitude AMF had lower viability rates. This means that the deleterious effects of IOMNP-MH are not related only to the macroscopic temperatures reached during MH, but are also associated with the amplitude of AMF. We interpreted this result through the occurrence of either some mechanical effects or by nanoscale (hot-spot) heating with the subsequent destruction of the endosomes/lysosomes in which they accumulate, suggesting that a further increase in IOMNP-MH efficiency might be obtained by specifically targeting the magnetic nanoparticles toward cell organelles that are more sensitive to nanoscale heating.

## Figures and Tables

**Table 1:** Structural information on MagNPs.

Sample	Amount of NaAc (g)	D_TEM_ (nm)	D_XRD_ (nm)	a (nm)
MagNP1	0.6	19.5 ± 0.23	19 ± 2.44	0.8371(1)
MagNP2	0.9	25 ± 0.32	23 ± 1.84	0.8376(3)
MagNP3	1.2	28.5 ± 0.15	25 ± 1.53	0.8379(2)
MagNP4	2.4	46 ± 0.18	42 ± 2.31	0.8387(9)
MagNP5	3.6	49.5 ± 0.15	45 ± 3.64	0.8385(7)
MagNP6	4.8	30.5 ± 0.3	30 ± 3.81	0.8380(2)

**Table 2:** Magnetic hysteresis parameters at room temperature for all six types of MagNPs.

Sample	M_s_ (emu/g)	H_c_ (kA/m)	M_r_ (emu/g)	M_r_/M_s_	K_eff_ (kJ/m^3^)
MagNP1	63	12.42	13.1	0.21	5.3
MagNP2	69	13.58	19.2	0.28	6.3
MagNP3	80	15.72	26.5	0.33	8.5
MagNP4	83	16.31	23.2	0.28	9.2
MagNP5	76	16.92	25.9	0.34	8.7
MagNP6	73	14.75	17.2	0.24	7.3

**Table 3:** Fitting parameters of SAR evolution with AC magnetic field amplitude (H).

Sample	Conditions	c (mg_Fe_/mL)	SAR_MAX_ (W/g_Fe_)	H_cHyp_ (kA/m)	Power Coefficient n
MagNP1	water	0.65	1350 ± 29.78	25.42 ± 0.61	3.24 ± 0.15
MagNP2			1840 ± 27.21	24.15 ± 0.39	3.31 ± 0.12
MagNP3			2260 ± 15.65	23.61 ± 0.17	4.74 ± 0.15
MagNP4			2280 ± 18.68	23.38 ± 0.21	4.85 ± 0.17
MagNP5			2030 ± 33.45	23.85 ± 0.42	3.72 ± 0.18
MagNP6			1580 ± 09.12	23.87 ± 0.15	3.68 ± 0.11
MagNP4	water	1.3	2390 ± 35.29	21.71 ± 0.38	4.93 ± 0.27
		0.65	2280 ± 18.68	23.38 ± 0.21	4.85 ± 0.17
		0.325	2380 ± 46.91	28.98 ± 0.51	3.90 ± 0.18
MagNP4	PEG8k-randomly distributed	1.3	1470 ± 22.68	29.51 ± 0.37	4.76 ± 0.21
		0.65	1940 ± 34.75	32.21 ± 0.44	4.41 ± 0.18
		0.325	2750 ± 74.51	32.71 ± 0.71	4.01 ± 0.21
		0.1625	3620 ± 99.05	35.76 ± 0.81	3.57 ± 0.13

## Supplementary Materials

The following are available online at https://www.mdpi.com/1999-4923/12/5/424/s1, Figure S1: Optical interference of IOMNPs with Alamar Blue and Neutral Red assays, Figure S2: Biochemical interference of IOMNPs with Alamar Blue and Neutral Red assays, Figure S3: Calibration curve used for the quantification of iron content of IOMNPs and the intracellular iron content, Figure S4: TEM images of magnetic nanoparticles synthesized with a very low amount of sodium acetate, Figure S5: Heating curves for the six types of IOMNPs dispersed in water, Figure S6: The dependence of SAR_MAX_ values of all six types of IOMNPs dispersed in water as a function of their saturation magnetizations, Figure S7: Heating curves for the MagNP4 dispersed in water, Figure S8: Heating curves for the MagNP4 dispersed in PEG8k, Figure S9: SAR dependence on H for MagNP4 dispersed in water and PEG8k, Figures S10 and S11: Heating curves of MagNP4 internalized in A549 and HGF, Figure S12: The SAR values of MagNP4 for three different values of H recorded in PEG8k and in both cells, Table S1: Amount of Fe^3+^ internalized in cells relevant for in vitro cytotoxicity assays and Fe^3+^ concentration of samples used in in vitro magnetic hyperthermia.
