# Supplementary material for: In Vitro Intracellular Hyperthermia of Iron Oxide Magnetic Nanoparticles, Synthesized at High Temperature by a Polyol Process
Source: Pharmaceutics. 2020 May 6;12(5):424. doi: 10.3390/pharmaceutics12050424 (PMC7285148; doi:10.3390/pharmaceutics12050424)
Supplement: Supplementary file 1 [file pharmaceutics-12-00424-s001.pdf]

pharmaceutics

Supplementary Materials: In Vitro Intracellular Hyperthermia of Iron Oxide Magnetic Nanoparticles, Synthesized at High Temperature by a Polyol Process

Cristian Iacovita, Ionel Fizeșan, Anca Pop, Lavinia Scorus, Roxana Dudric, Gabriela Stiufiuc, Nicoleta Vedeanu, Romulus Tetean, Felicia Loghin, Rares Stiufiuc and Constantin Mihai Lucaciu

1. Optical and biochemical interference of IOMNPs with Alamar Blue and Neutral Red assays.

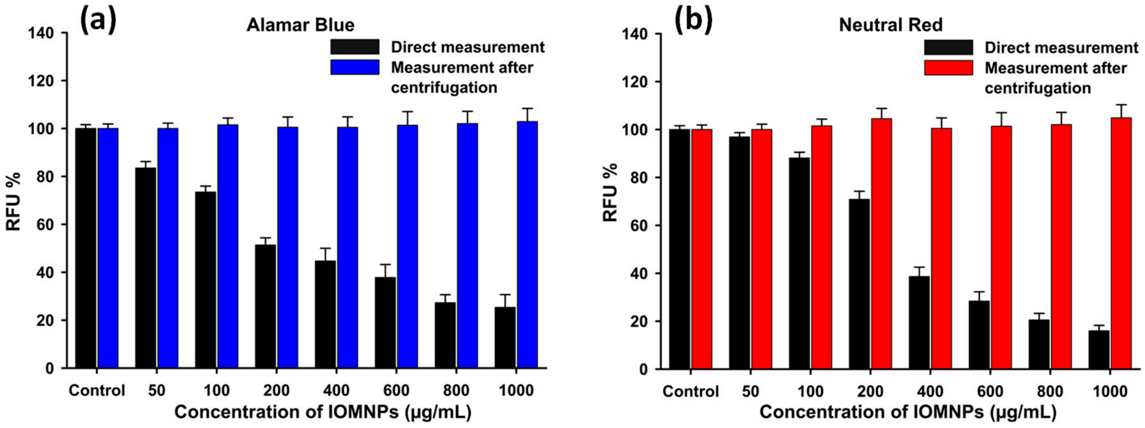

Figure S1. Optical interference of IOMNPs with Alamar Blue assay (**a**) and Neutral Red assay (**b**). Different quantities of IOMNPs were extempore mixed with resorufin and neutral red and the fluorescence was measured directly or after a centrifugation step. The values are expressed as mean ± SD of three replicates. Data were expressed as relative values to the negative control.

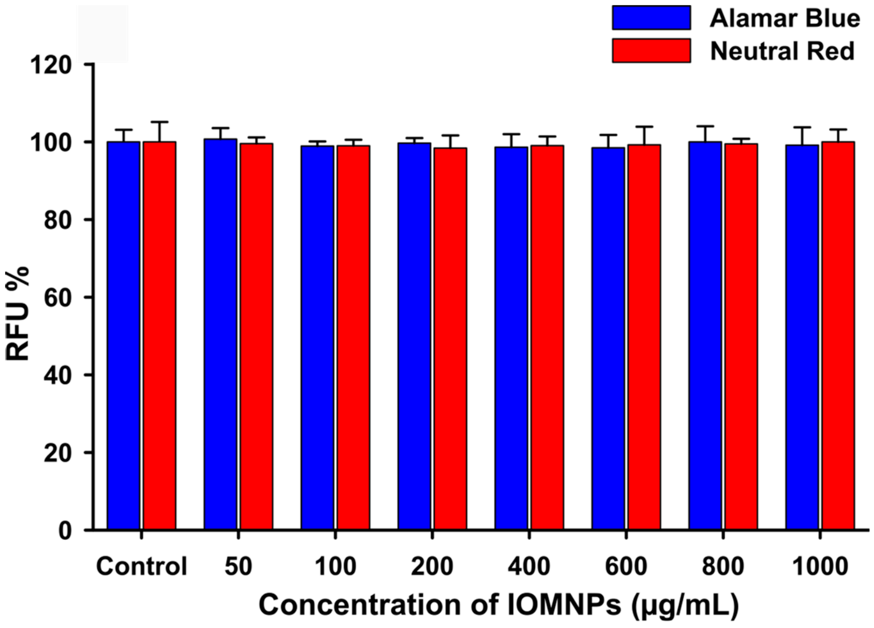

Figure S2. Biochemical interference of IOMNPs with Alamar Blue assay and Neutral Red assay. Different quantities of IOMNPs were incubated with resorufin and neutral red for 3 hours. After the incubation, all samples were centrifuged and the fluorescence was measured. The values are expressed as mean ± SD of three replicates. Data were expressed as relative values to the negative control.

2. Calibration curve used for the quantification of the iron content of IOMNPs and the intracellular iron content.

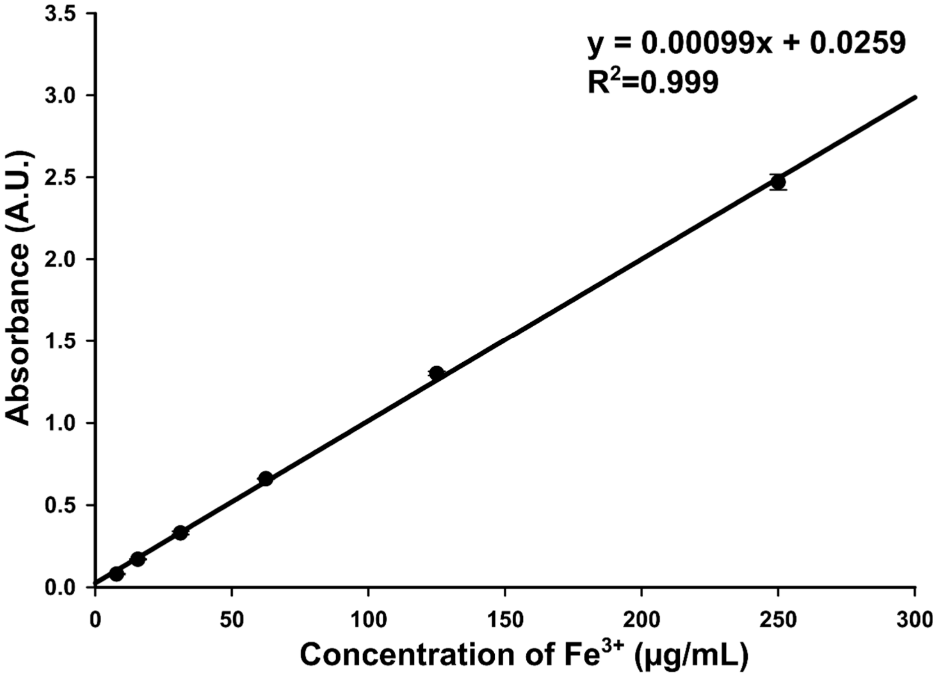

Figure S3. The absorbance of six standard Fe^{3+} colloidal solutions as a function on Fe^{3+} concentration measured at a λ = 490 nm. The values are expressed as mean ± SD of three replicates. The black line represents a linear regression of the experimental values.

3. TEM images of magnetic nanoparticles synthesized with a very low amount of sodium acetate

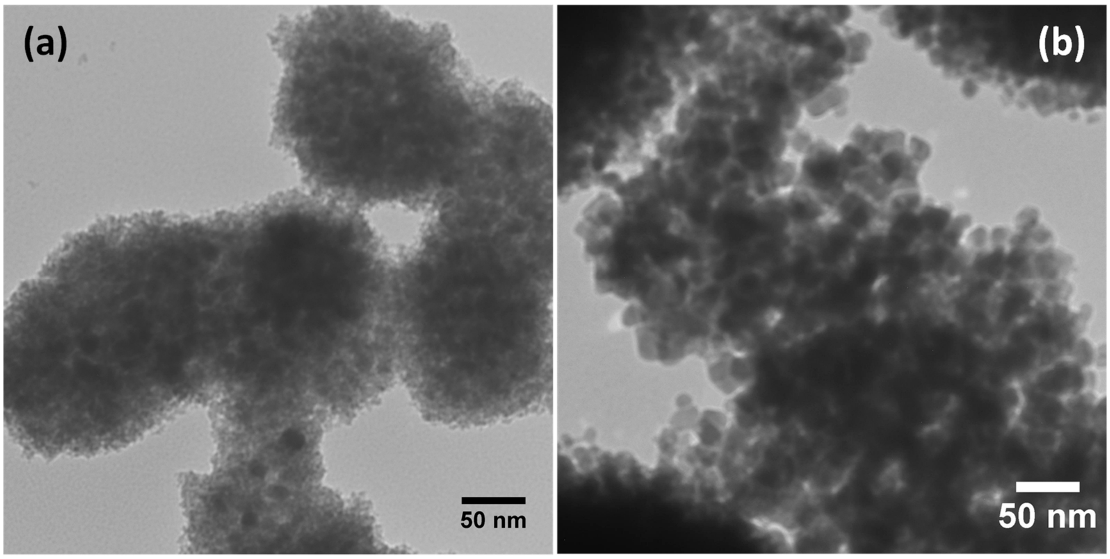

Figure S4. Large scale TEM images of MagNPs obtained by employing different amounts of NaAc in the synthesis: (**a**) 0.1 g and (**b**) 0.3 g.

4. Heating curves—T = f (time) curves—for the six types of MNPs dispersed in water

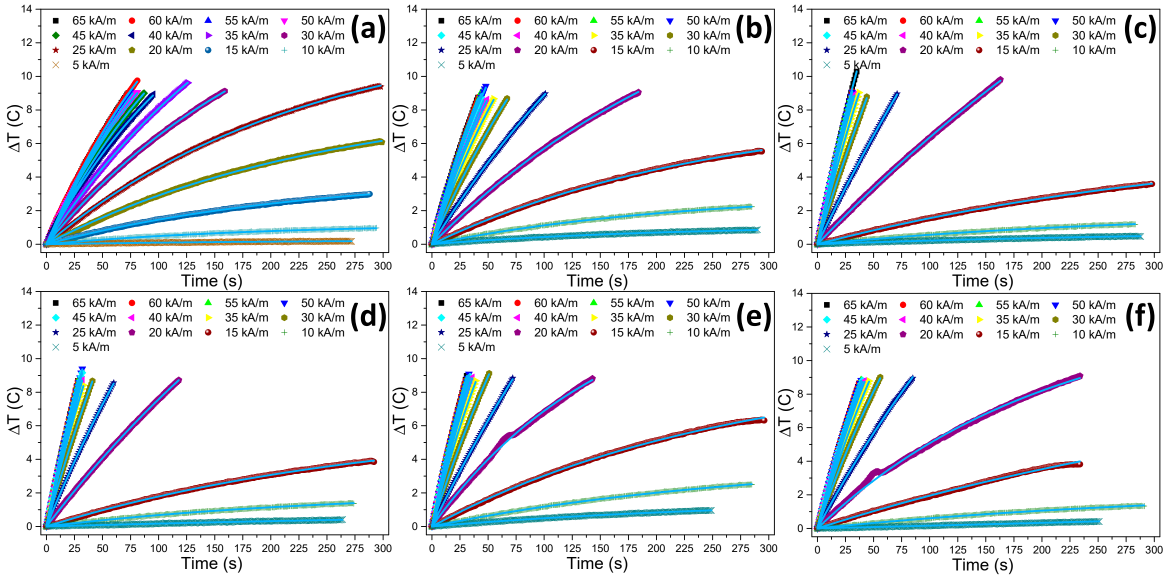

Figure S5. Heating curves fitted with Box-Lucas equation (blue curves) of (**a**) MagNP1, (**b**) MagNP2, (**c**) MagNP3, (**d**) MagNP4, (**e**) MagNP5 and (**f**) MagNP6 dispersed in water at a concentration 0.65 mg_{Fe}/mL, recorded as a function of AC magnetic field amplitudes at frequency of 355 kHz.

5. The dependence of SAR_{MAX} values as a function of the saturation magnetizations.

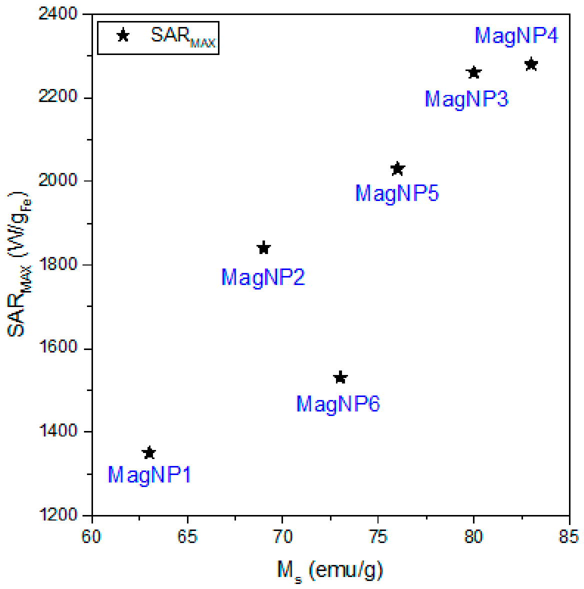

Figure S6. The SAR_{MAX} values of MagNPs dispersed in water as a function of their saturation magnetizations.

6. Heating curves—T = f(time) curves—for the IOMNPs from sample MagNP4 dispersed in water at different concentration of iron content

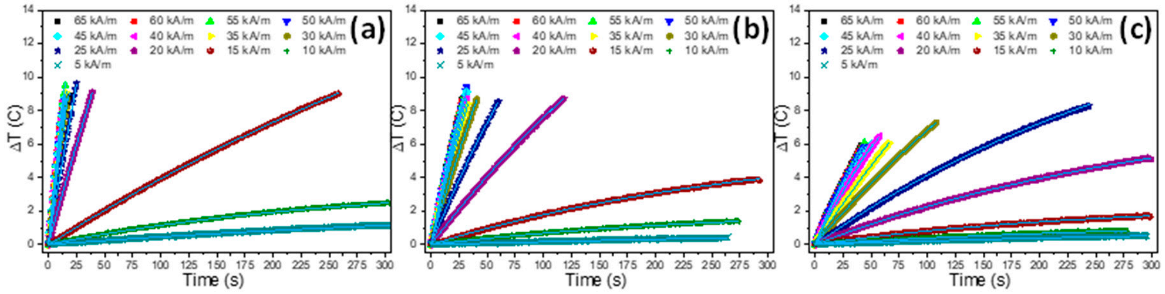

Figure S7. Heating curves fitted with Box-Lucas equation (blue curves) of MagNP4 dispersed in water at concentrations of (**a**) 1.3 mgFe/mL, (**b**) 0.65 mgFe/mL and (**c**) 0.325 mgFe/mL recorded as a function of AC magnetic field amplitudes at frequency of 355 kHz.

7. Heating curves T = f(time) curves for the IOMNPs from sample MagNP4 dispersed PEG8K at different concentration of iron content

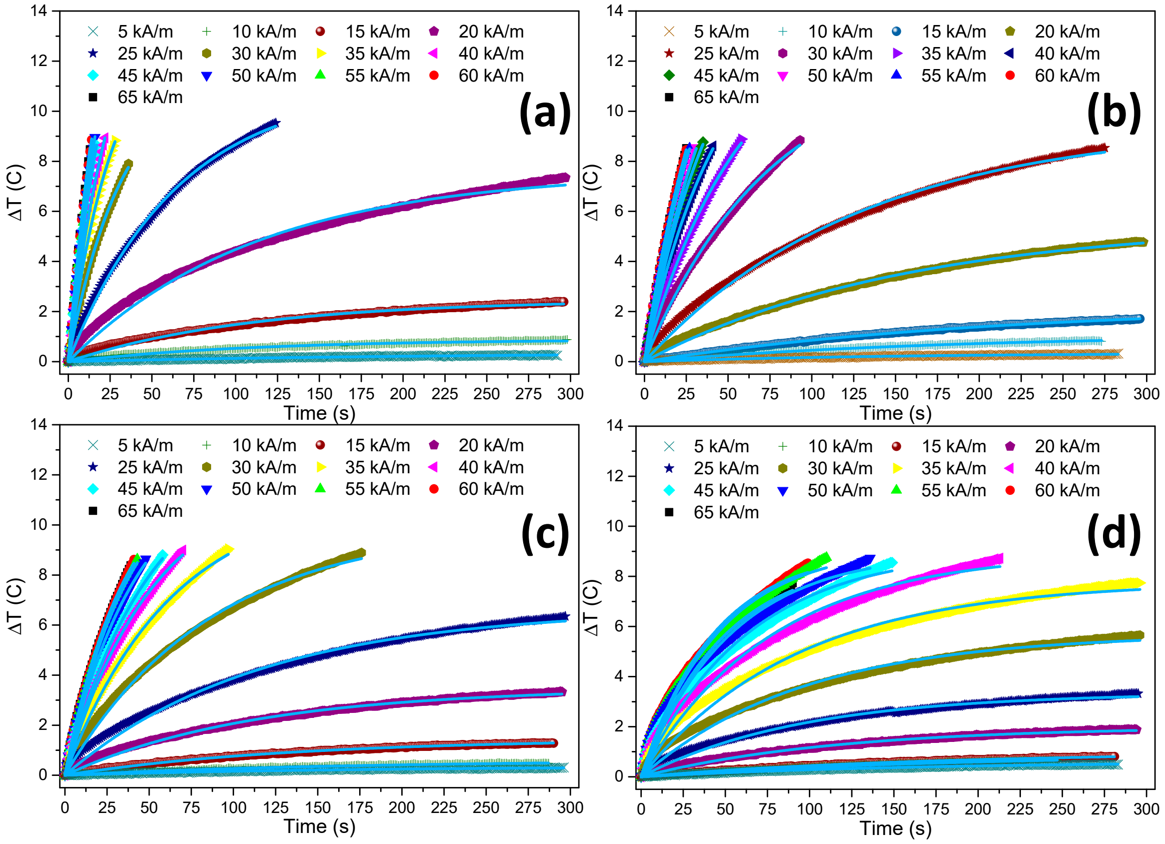

Figure S8. Heating curves fitted with Box-Lucas equation (blue curves) of MagNP4 dispersed in PEG8k at concentrations of (**a**) 1.3 mgFe/mL, (**b**) 0.65 mgFe/mL (**c**) 0.325 mgFe/mL and (d) 0.1625 mgFe/mL recorded as a function of AC magnetic field amplitudes at frequency of 355 kHz.

8. Comparison between SAR values recorded in water and PEG8k at different concentrations of MagNP4 as a function of H.

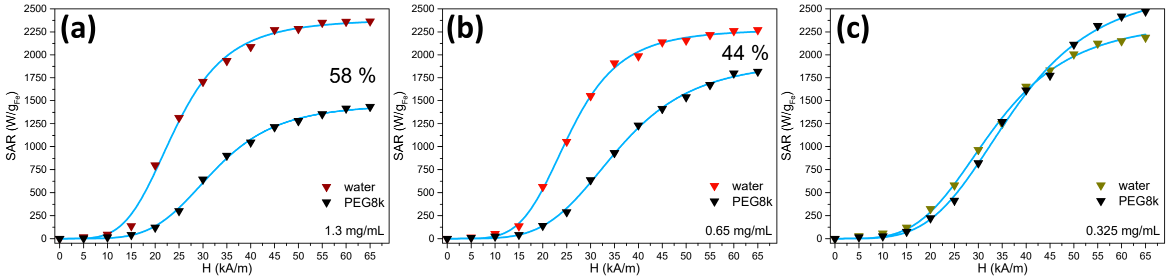

Figure S9. SAR dependence on H for MagNP4 dispersed in water and PEG8k at an iron content of (**a**) 1.3 mg_{Fe}/mL; (**b**) 0.65 mg_{Fe}/mL and (**c**) 0.325 mg_{Fe}/mL. The blue lines represent the fits with the logistic function.

9. Heating curves T = f(time) curves for the IOMNPs from sample MagNP4 internalized in A549 and HGF cells at different concentration of iron content

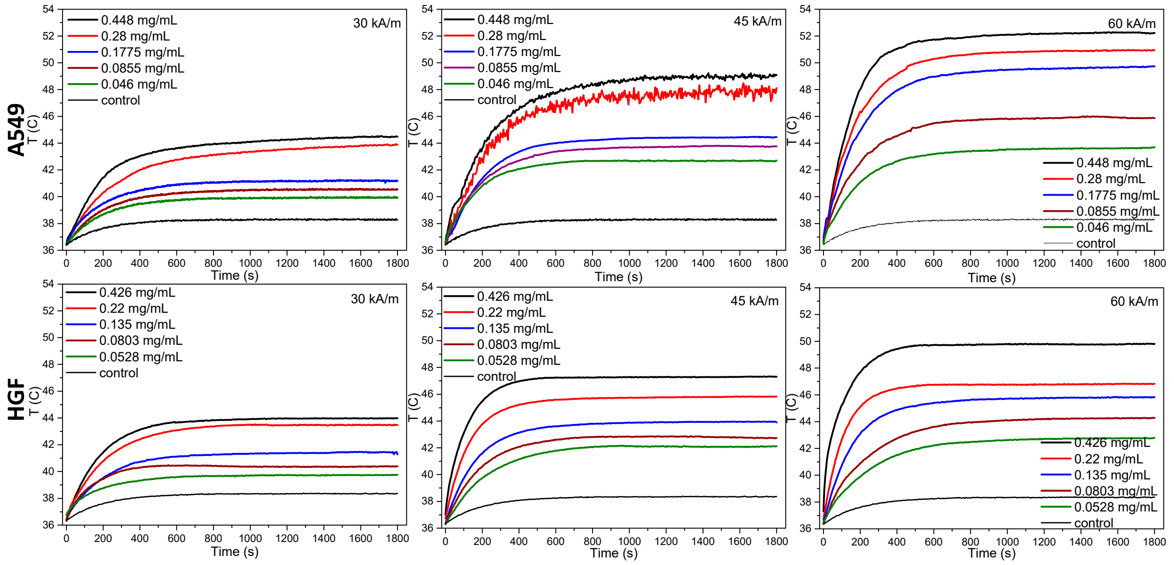

Figure S10. Heating curves of MagNP4 internalized in A549 (upper panels) and HGF (lower panels) at different concentration of iron content recorded in a volume of 200 µL at different H values: 30 kA/m (left panels), 45 kA/m (middle panels) and 60 kA/m (right panels) at a constant frequency of 355 kHz.

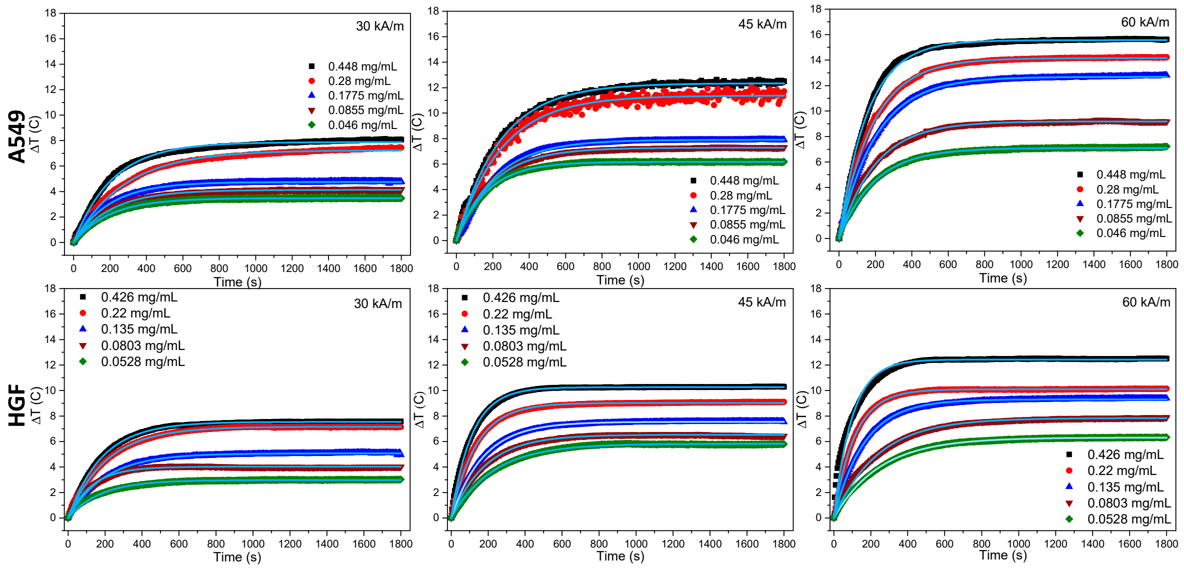

Figure S11. Heating curves fitted with Box-Lucas equation (blue curves) of MagNP4 internalized in A549 (upper panels) and HGF (lower panels) at different concentration of iron content recorded in a volume of 200 μL at different H values: 30 kA/m (left panels), 45 kA/m (middle panels) and 60 kA/m (right panels) at a constant frequency of 355 kHz.

10. Cellular uptake of IOMNPs

According to sub-section *2.7. Evaluation of Cellular Uptake* from the main text the cells were exposed to 2 mL of colloidal solution of IOMNPs dispersed in the cellular medium at increasing concentration from 50 to 1000 μg_{IOMNPs}/mL. Based on the thiocyanate assay described in sub-section *2.6. Iron concentration determination* and a standard curve representing the absorbance of the trivalent iron ions colloidal solutions as a function on their concentration (Fig. S11), the *intracellular iron content was determined. As described in sub-section* 2.8. In vitro magnetic hyperthermia, following the washing and trypsinization steps, the cells were dispersed in 1 mL of cell culture media. Afterward, two equally aliquots of 400 μL each were separated, the cells were gently centrifuged and 200 μL of cell culture media was removed from each aliquot. Consequently, the iron concentration of the samples, containing IOMNPs loaded cancer cells, exposed to AMF (the last column from table S1) has been calculated by dividing the 40% of the internalized amount of Fe^{3+} ions to 0.2 mL (200 μL).

Table S1. Amount of Fe^{3+} internalized in cells relevant for in-vitro cytotoxicity assays and Fe^{3+} concentration of samples used in *in vitro* magnetic hyperthermia.

Cell	Exposed Dose of IOMNPs (μg /mL)	Exposed Amount of IOMNPs (μg)	Internalized Amount of IOMNPs (μg)	Internalized Amount of Fe^{3+} Ions (μg)	In Vitro Magnetic Hyperthermia Concentrations (μg_{Fe} /mL)
A549	1000	2000	344.82	224.13	448.3
	500	1000	215.78	140.25	280.1
	250	500	136.55	88.75	177.5
	100	200	65.80	42.77	85.5
	50	100	35.40	23.01	46.2
HGF	1000	2000	328.08	213.25	426.2
	500	1000	169.43	110.13	220.1
	250	500	103.61	67.34	135.4
	100	200	61.79	40.16	80.3
	50	100	40.60	26.39	52.8

11. Comparison between SAR values recorded in PEG8k and in both cells at almost similar concentrations of MagNP4 as a function of H.

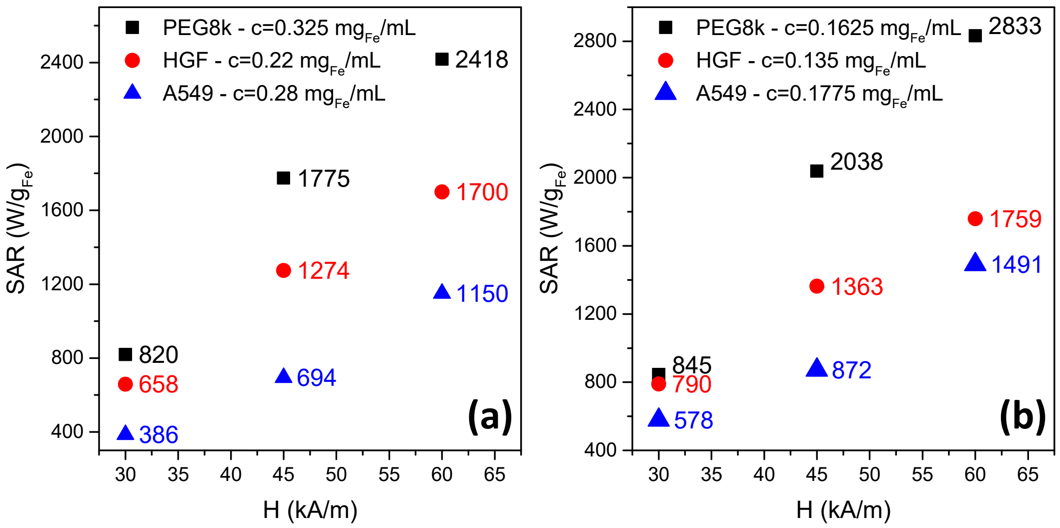

Figure S12. The SAR values of MagNP4 for three different values of H (30, 45 and 60 kA/m) recorded in PEG8k and in both cells at concentration around (**a**) 0.325 mg_{Fe}/mL and (**b**) 0.1625 mg_{Fe}/mL.
